# Controlling cantilevered adaptive X-ray mirrors

**DOI:** 10.1107/S1600577524006234

**Published:** 2024-08-05

**Authors:** Kenneth A. Goldberg, Kyle T. La Fleche

**Affiliations:** a Advanced Light Source Lawrence Berkeley National Laboratory Berkeley CA94720 USA; b Engineering Division Lawrence Berkeley National Laboratory Berkeley CA94720 USA; Tohoku University, Japan

**Keywords:** adaptive optics, X-ray optics, mirrors, wavefront control, mounting

## Abstract

Modeling the behavior of a prototype cantilevered X-ray adaptive mirror (held from one end) demonstrates its potential for use on high-performance X-ray beamlines. Similar adaptive mirrors are used on X-ray beamlines to compensate optical aberrations, control wavefronts and tune mirror focal distances at will. Controlled by 1D arrays of piezoceramic actuators, these glancing-incidence mirrors can provide nanometre-scale surface shape adjustment capabilities. However, significant engineering challenges remain for mounting them with low distortion and low environmental sensitivity. Finite-element analysis is used to predict the micron-scale full actuation surface shape from each channel and then linear modeling is applied to investigate the mirrors’ ability to reach target profiles. Using either uniform or arbitrary spatial weighting, actuator voltages are optimized using a Moore–Penrose matrix inverse, or pseudoinverse, revealing a spatial dependence on the shape fitting with increasing fidelity farther from the mount.

## Abstract 2

The linear shape control and actuation of a modeled adaptive X-ray optic in a cantilever geometry is described, showing that control is mathematically similar to that of the more common mounting approaches.

## 1. Introduction

X-ray free-electron laser and synchrotron light sources with high coherent flux place high demands on the quality of beamline optics, including X-ray mirrors (Cocco *et al.*, 2022 ▸). Preserving and controlling X-ray wavefronts requires atomically smooth glancing-incidence mirrors with nm or sub-nm surface figure quality. On beamlines with multiple elements, an adaptive X-ray optic (AXO) can provide a means to correct wavefront aberrations from inherent mirror shape errors, misalignment, thermo-mechanical drift, distortion from power loading and mechanical instabilities (Susini *et al.*, 1995 ▸; Sutter *et al.*, 2022 ▸; Gunjala *et al.*, 2023 ▸; Rebuffi *et al.*, 2023 ▸).

On beamlines where the control of coherent X-ray light is important, beams typically measure not more than a few mm wide. To achieve high reflectivity, X-ray mirrors operate in glancing incidence, with angles below 2°. Beam footprints on mirror surfaces are sagitally narrow and tangentially long (hundreds of mm is common). For this practical reason, AXOs usually bend only along the tangential (meridional) direction of the beam footprint. For stability, the substrates used for adaptive mirrors are commonly several cm thick and wide.

Mechanically bendable X-ray optics have been used for many decades, providing static or dynamic 1D focusing (Underwood & Turner, 1977 ▸; Howells, 1995 ▸; Padmore *et al.*, 1996 ▸). Many mirror benders are limited to correcting curvature (second-order) and coma (third-order) (Howells *et al.*, 2000 ▸).

More sophisticated adaptive X-ray mirrors have been studied since the 1990s. Pioneering demonstrations (Susini *et al.*, 1995 ▸, 1996 ▸; Signorato *et al.*, 1998 ▸) showed that piezo-bimorph actuators could be applied. Since that time, fabrication and control have improved through several generations. Typically, AXOs create local curvature across a number of discrete, addressable channels. This has led to demonstrations of arbitrary shapes (Sawhney *et al.*, 2010 ▸; Alcock *et al.*, 2015 ▸; Sutter *et al.*, 2016 ▸), rapid shape correction (Alcock *et al.*, 2023 ▸) and nm-scale dynamic control (Gunjala *et al.*, 2023 ▸). Feedback from *in situ* shape measurement (Alcock *et al.*, 2023 ▸) or at-wavelength techniques (Yumoto *et al.*, 2006 ▸; Goldberg *et al.*, 2021 ▸; Frith *et al.*, 2023 ▸) is essential for successful, practical AXO systems.

Researchers have devised several different approaches to mechanically support and bend AXO mirrors. There are mirrors with segmented arrays of bonded piezoceramic elements (Alcock *et al.*, 2015 ▸; Ichii *et al.*, 2019 ▸), and others with springs and motors (Colldelram *et al.*, 2017 ▸). Mechanical holders may contact the mirror at its Airy or Bessel points to minimize the mounting’s influence on the surface shape (Smith & Chetwynd, 2005 ▸). When clamps are used, careful engineering is required to balance the need to secure the mirror with the need for compliance. In all cases, care must be taken to minimize twisting stresses that can be induced by certain mounts (Alcock *et al.*, 2019 ▸).

In this context, *cantilevered*X-ray mirrors, held only from one side, are far less common. This approach may be impractical for longer mirrors (exceeding 500 mm) due to limited stiffness, gravitational sag and low first-resonance frequencies making them vulnerable to vibration. Yet, for shorter mirrors, we contend that cantilevered mounting conveys significant advantages, including their use as AXOs. Here we describe the linear shape control and actuation of a modeled AXO in a cantilever geometry. Our goal is to show that control is mathematically similar to that of the more common mounting approaches.

## 2. Modeling the cantilever AXO

**Figure 1:**
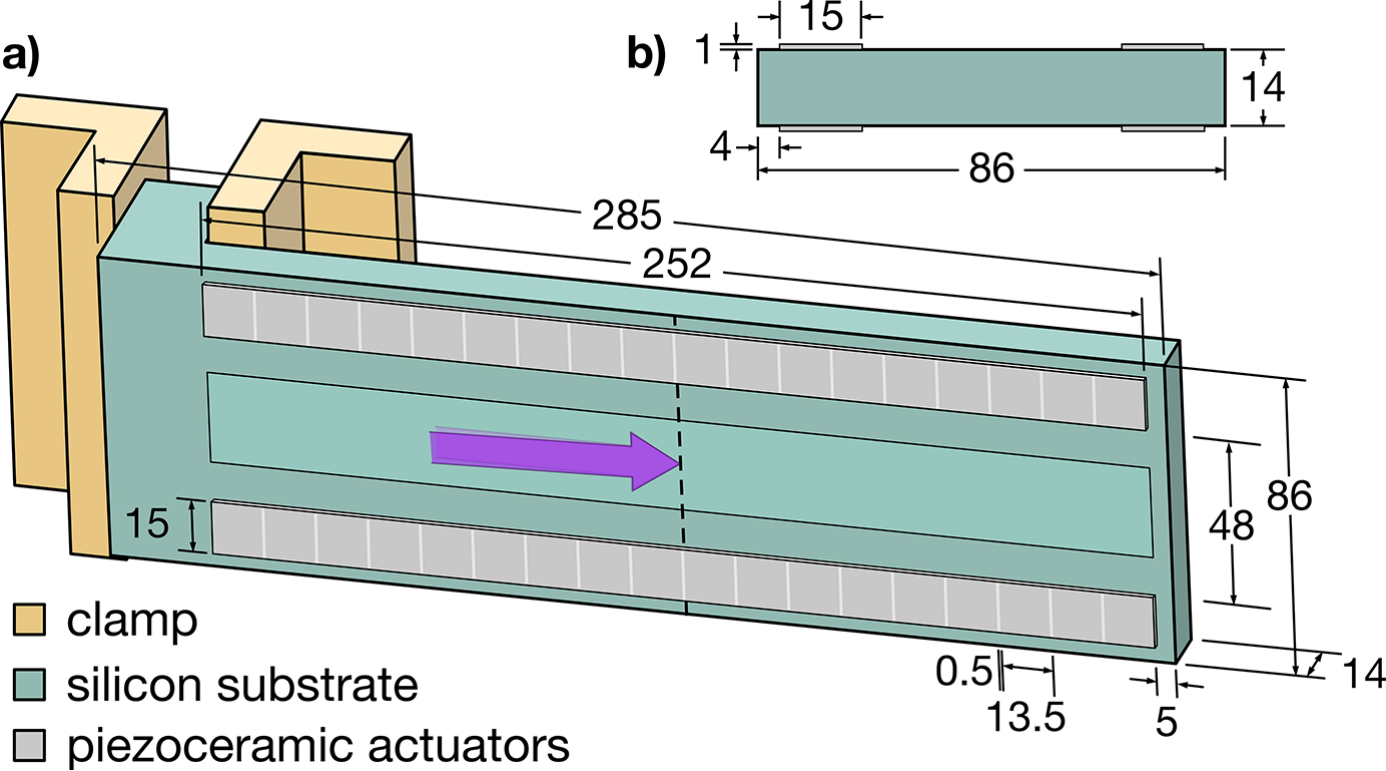
(*a*) Model geometry for the cantilevered adaptive mirror held for horizontal beam deflection. Clamping on the right-angled portion of the L-shaped substrate is intended to minimize surface distortion. The dashed line indicates the axis of *pitch* rotation, passing through the central ray of the incident beam footprint. The magenta arrow shows the incident light direction. (*b*) A cross section shows the positions of the four symmetrically placed piezoceramic elements. All units are mm.

We used *Ansys* software (Ansys, 2022 ▸) to create and study a finite-coupled-field static analysis model of a prototype cantilever AXO mirror with finite-element analysis (FEA). The mirror is modeled with the geometry and dimensions shown in Fig. 1 ▸. With the mirror mounted for horizontal beam deflection, the small effects of gravity, pulling the mirror in its sagittal direction, were neglected. The material properties are listed in Table 1 ▸.

The optical geometry is intended for a beam incident at a 1.25° glancing angle of incidence. The tangential beam footprint can be up to 252 mm long, and the clear-aperture width on the mirror is 28 mm. The silicon substrate is L-shaped, comprising a long, unsupported mirror region, and a shorter perpendicular section clamped to the mount as shown. This design is intended to minimize the influence of the clamping on the mirror surface shape while allowing the mirror actuators to bend freely. The 14 mm mirror substrate thickness was chosen as a compromise between stiffness and bending flexibility.

The four piezoceramic elements form a symmetric sandwich on the front and back sides that maintains an unobstructed, central clear aperture. The symmetric arrangement promotes temperature stability, balancing the forces of differential thermal expansion (Ichii *et al.*, 2021 ▸). The actuators are divided longitudinally into 18 separate channels, wired to provide equal and opposite bi-directional bending forces (lengthwise contraction and expansion) from the front and back sides. The channels are 13.5 mm long with a 0.5 mm gap, creating a 14 mm spatial period. In this study, the actuator closest to the clamp is not used, leaving 17 active, individually addressable channels. Actuation with applied voltages up to ±500 V induces concave or convex local bending.

The FEA model leverages a previous, related study conducted by the authors (Goldberg *et al.*, 2021 ▸). The response of the Fujicera C-213 piezoceramic material layers is described by Fujicera (2022 ▸).

It is important to bear in mind that the required surface deflection for X-ray mirrors ranges from nm to µm, and that the relevant size of optical aberrations that must be controlled to achieve diffraction-limited performance is typically on the order of several nm. Furthermore, the adaptive optic is designed to address or compensate only low-spatial-frequency wavefront changes in 1D. In this analysis, we neglect the anisotropic properties of silicon which would affect sagittal bending.

**Figure 2:**
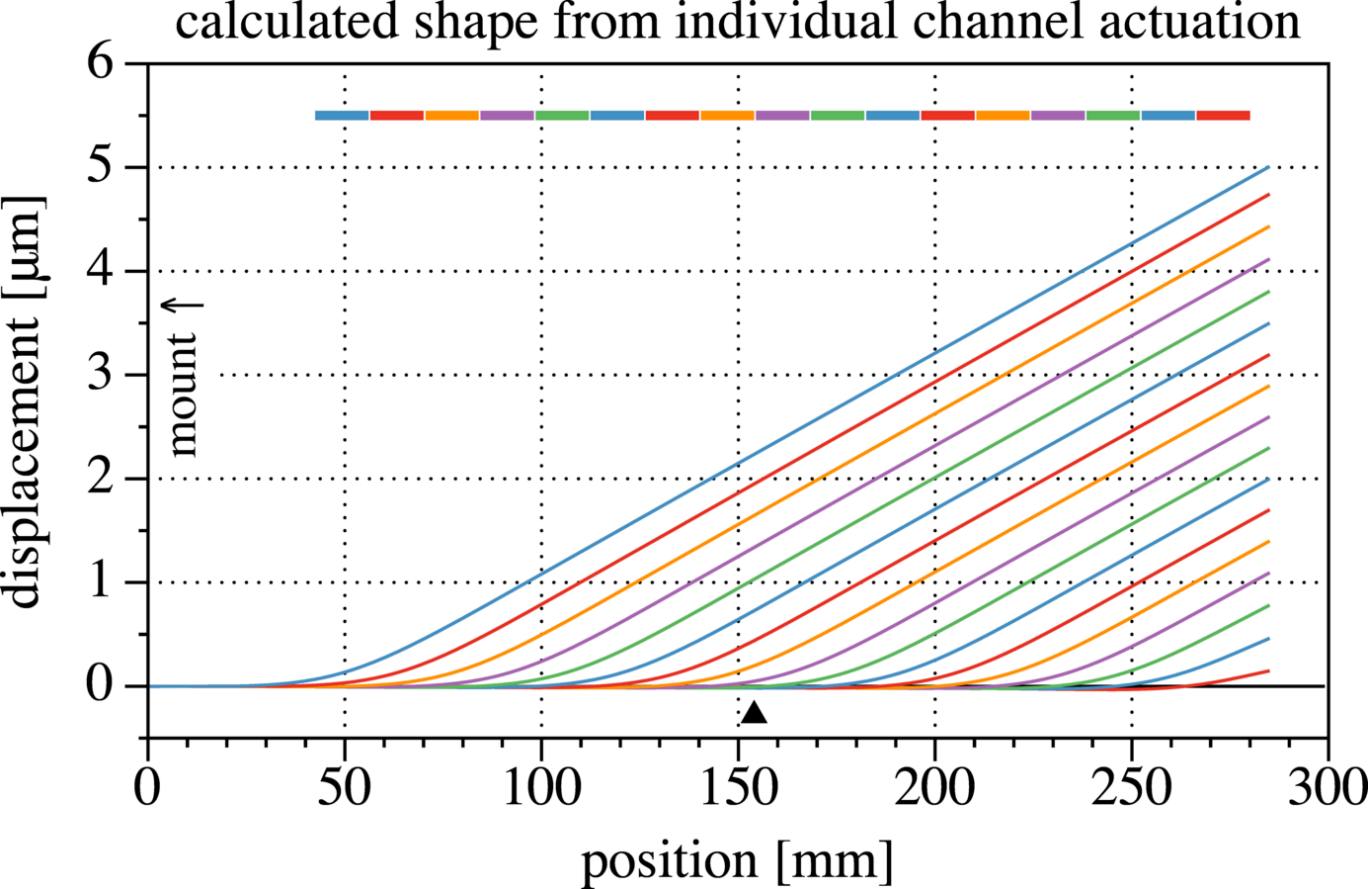
Surface shapes calculated with +500 V applied to the actuators of each channel individually. The mirror is pinned by its mount at the *x* = 0 position. In this and subsequent plots, actuator locations and sizes are represented by the line segments above the surface data. The black triangle marks the longitudinal center of the pitch rotation.

With the mirror clamped on the upstream side (stationary, with zero slope at *x* = 0), Fig. 2 ▸ shows the shapes resulting from +500 V actuation of each individual channel. The surface shape was extracted from the model on a 1 mm grid.

**Figure 3:**
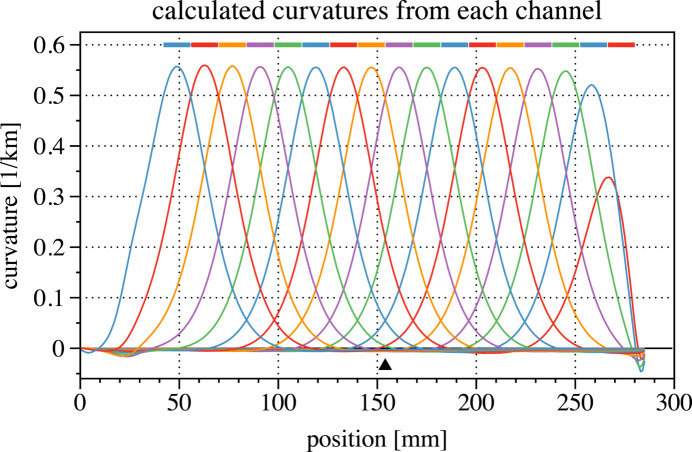
Curvature of the surface shapes shown in Fig. 2 ▸. With +500 V applied to individual channels the local radius of curvature is below 2 km.

The calculated second derivatives, related to the local curvature, are shown in Fig. 3 ▸. While each actuation channel bends the mirror across the region of that actuator, the curvature plot shows that the influence extends nearly 50 mm in each direction from its center. The model also predicts a relatively small amount of reverse bending from the regions to the left and right of the channel. Reversing the sign of the applied voltage produces inverted versions of these shapes.

In our model, the prototype mirror mount has a tilt actuator that applies *pitch* rotation about the light’s central-ray intersection point (*i.e.* the center of the beam footprint) when the mirror is in its rest position. This axis is 154 mm from the clamped end of the mirror, in the center of the actuated region, and is indicated by a dashed line in Fig. 1 ▸. However, the mount cannot displace the mirror perpendicular to its surface to accommodate the small position offsets that arise from bending. Without this tilt degree of freedom, the actuator farthest from the free end could be used for tilt control; however, as discussed, it is coupled to the displacement of the mirror center.

The lack of displacement is not a drawback of this design, it is a simplification of the required mechanism. This approach works for two reasons. First, the displacements (less than 5 µm, as shown in Fig. 2 ▸) are a small fraction of the beam width, so the tangential beam position on the mirror does not shift significantly. Second, the pitch angle change required to compensate for this µm-scale displacement (that is, to stabilize the beam at a point several meters downstream of the mirror) is much less than 1 µrad. For example, to compensate a 2 µm mirror displacement and stabilize the beam position 4 m downstream of the mirror, the mirror tilts by 250 nrad. With this small change of pitch, the ends of the mirror move by less than 40 nm. Thus the beam shape correction and pointing stability can be included in the programmed actuation in the cantilevered mirror and mount.

## 3. Linear solution model

Setting aside the hysteretic and the dynamic behaviors of piezoceramic actuators (creep, drift *etc*.) (Alcock *et al.*, 2019 ▸; Gunjala *et al.*, 2023 ▸), a linear actuation model provides a helpful understanding of the ideal system performance when trying to achieve arbitrary static shapes. This approach follows similar shape optimization work in X-ray optics with the method of *characteristic functions* (Hignette *et al.*, 1997 ▸; Signorato *et al.*, 1998 ▸; McKinney *et al.*, 2009 ▸) and with linear-response modeling of AXOs (Signorato *et al.*, 1998 ▸; Idir *et al.*, 2010 ▸). Solution with or without displacement as a free parameter proceeds as follows.

### 3.1. Solution with displacement

To achieve a desired mirror surface shape, we apply the Moore–Penrose matrix inverse (also called the pseudoinverse) (Moore, 1920 ▸; Penrose, 1955 ▸; Lawson & Hanson, 1995 ▸) to guide the optimization of actuator voltages, mirror tilt and displacement (when displacement is available as a free parameter). Like the method of characteristic functions, this approach minimizes the squared difference calculated point-by-point, with either uniform or non-uniform weightings, as required. It is a direct, non-iterative solution.

We build a matrix **A** containing, as columns, the mirror shape resulting from the actuation of each individual channel, measured either from the zero-voltage, *at-rest* position or as a relative shape change (*i.e.* the point-by-point difference), per unit of applied voltage change. Additional columns can be added for (i) constant shape offset (if that is available as a degree of freedom) and (ii) for the linear pitch (*i.e.* tilt) by the mirror mount about the central point of intersection with the beam. Using 17 actuator channels plus the one or two additional degrees of freedom, the matrix has 18 or 19 columns. The surface descriptions make the matrix 285 rows tall. The constant offset column, if present, should have uniform values defined in height units that are relevant to the positioning control. Similarly, the tilt column can be a linear ramp passing through zero at the center of mirror pitch rotation. This represents a µrad or nrad rotation that the actuator produces.

A target shape, **b**, is represented as a vector of 285 points. The pseudoinverse approach solves the set of homogeneous linear equations (**Ax** ≃ **b**) similar to singular-value decomposition (SVD) (Strang, 1988 ▸). The solution 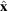 that minimizes the square difference, calculated as the discrete sum over all surface points, 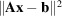, is 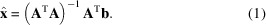
The pseudoinverse matrix is characteristic of the actuation parameter space and only needs to be calculated once for the system, including the weighting. The columns of **A** can be calculated with FEA modeling or measured *in situ* with the actuation of each individual channel in turn.

### 3.2. Solution without displacement

**Figure 4:**
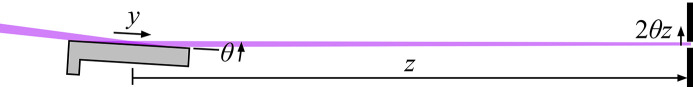
Downstream beam position stabilization in the presence of mirror tilt and position offset requires the coupling of the two terms in our solution. *y* is the tangential position along the surface. *z* is the distance from the mirror center to the aperture where stabilization is required. θ is not the incident angle but the mirror pitch angle relative to its optimal position.

In some beamline optical systems where adaptive mirrors are used, maintaining beam stabilization and fine alignment on a downstream aperture (*e.g.* an exit slit) is an important requirement. As described above, our prototype mirror mount does not offer independent lateral positioning as part of routine, fast, mirror surface control. We therefore apply small pitch rotations to maintain stable alignment on a fixed exit slit *z* = 4 m downstream of the mirror center. Prioritizing the downstream beam position in this way requires us to couple the position offsets of the shape actuation to the mirror tilt that we apply. To first approximation, the downstream position is controlled by the mirror tilt angle. It moves with 2θ*z*: the ray deflection angle caused by mirror tilt, 2θ, multiplied by the distance to the aperture, *z*, as shown in Fig. 4 ▸.

In this solution, the matrix **A** contains 17 columns for the surface actuations and one column for the *combined tilt and displacement*, labeled **t**. With **y** as a column vector that describes the position along the mirror, **t** is 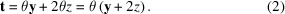
The column vector **t** contains the mirror surface tilt and the constant downstream beam displacement that comes from the angle change. Optimizing this degree of freedom with the others stabilizes the beam position on the downstream aperture in the presence of mirror surface displacement. Here, the matrix **A** has 18 columns and 285 rows, and the solution for a target surface shape **b** follows equation (1), as before.

### 3.3. Weighted solution to optimize the Strehl ratio

The surface shape optimization described above assigns uniform importance (weighting) to every point in the fitting domain. For a host of physical reasons, this approach may not produce optimal beam focusing or control. For example, optimizing the peak intensity at the focus of a coherent beam with a non-uniform intensity profile across the aperture requires a physical weighting that matches the amplitude. This and other physical considerations are described by Goldberg & Yashchuk (2016 ▸). Therefore, whether optimizing the Strehl ratio, accounting for varying distance from the focus along the mirror, accounting for spatially varying reflectivity and phase, or considering any other physical parameters, we should include spatial weighting in the optimization.

There are several solutions for the problem with non-uniform weights. Goldberg & Yashchuk (2016 ▸) apply the weighting using the method of least-squares as derived by Bevington & Robinson (2003 ▸). The general regression approach was given by Yashchuk (2006 ▸), with and without weighting. The approach with the method of characteristic functions was described by McKinney *et al.* (2009 ▸).

The SVD solution has been generalized (Van Loan, 1976 ▸) and weighted solutions have been given by numerous authors, including Nikolaevskaya & Khimich (2009 ▸), Sergienko & Galba (2015 ▸) and Jozi & Karimi (2018 ▸). We adapt this weighting to the pseudoinverse approach as follows. Given a vector of weights across the surface, **w**, we minimize the weighted square difference written as 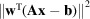 or 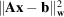. To solve, we create the diagonal square matrix **W** from diag(**w**^1/2^), then replace **A** with 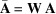 and **b** with 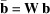 in equation (1), 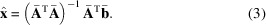
In this way, the resultant 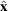 vector optimizes the voltages and stage tilt for the *original***A** basis using the weighted sum 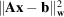. (Equivalently, it optimizes 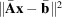.)

## 4. Demonstrations

Three case studies demonstrate the application of this approach to the cantilevered-mirror geometry. These arbitrary target surface shapes represent possible configurations for wavefront-error compensation, for changing the focal plane position or for imparting intentional phase variation across the wavefront.

Target shapes were generated in two ways. The first, Shape 1, is a Gaussian surface bump profile, centered on the mirror’s central point of intersection, with 100 mm full width at half-maximum (FWHM), and normalized to have an r.m.s. amplitude of 10 nm across the domain. This would be considered a relatively easy shape to fit. The second two profiles, Shapes 2 and 3, were created from a series of the first 15 Legendre polynomials, with the constant and the linear terms set to zero. The coefficients of the individual terms were randomly chosen on a domain from −1 to 1, and then scaled by the reciprocal of the order number to reduce the amplitude of the higher-ordered terms. The surfaces were normalized to have an r.m.s. amplitude of 10 nm across the domain. Before normalization, a multiplicative filter was applied to reduce the amplitude to zero at the two endpoints where there are no piezoactuators.

**Figure 5:**
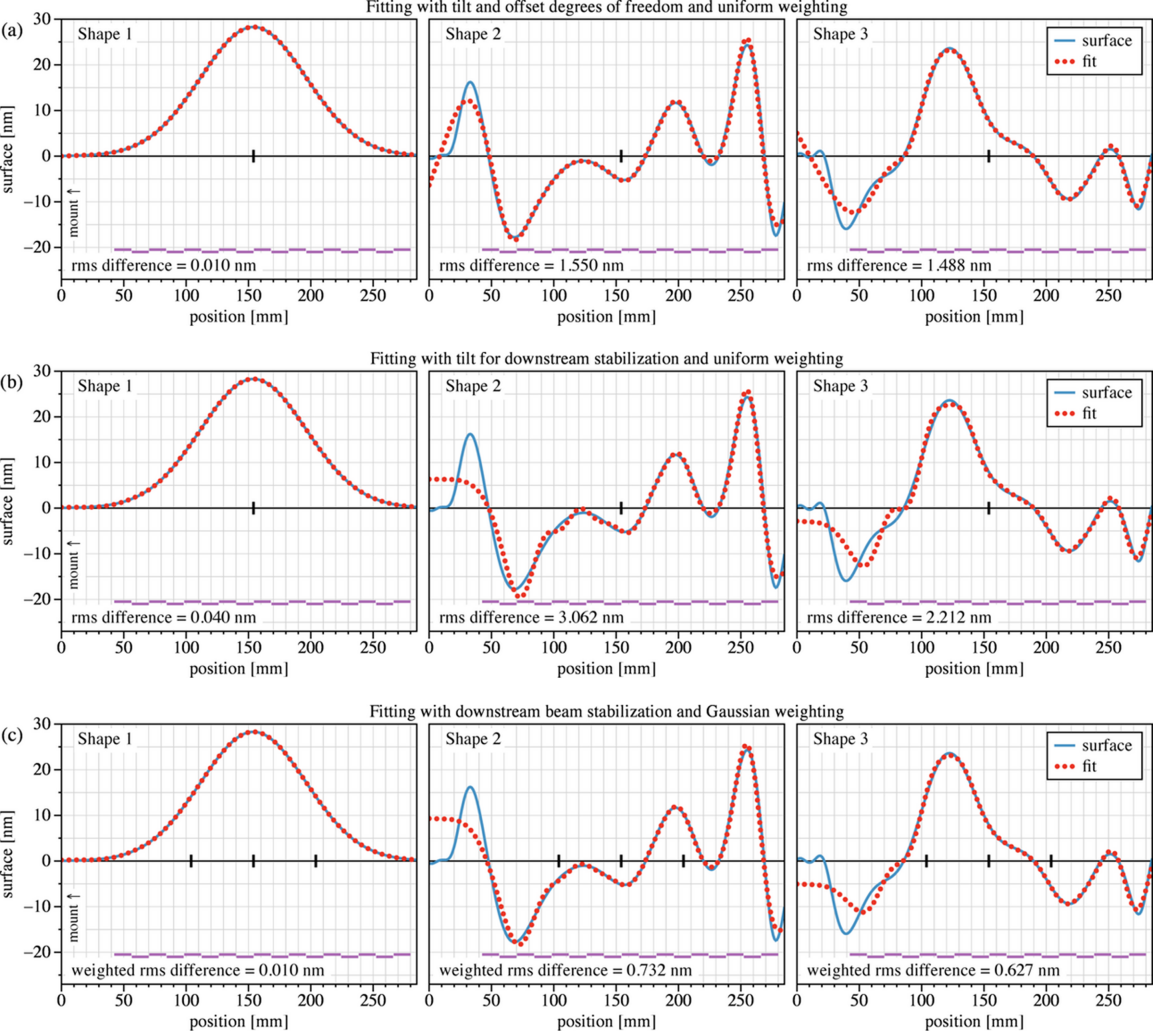
Three target surface shapes are fitted using the methods described herein. (*a*) Fitting is applied with 17 actuators plus constant and linear terms, and uniform weighting across the domain. (*b*) The constant term is removed and the linear term is replaced with tilt for beam stabilization downstream. (*c*) The mirror actuation of (*b*) is applied with Gaussian *amplitude* weighting. The vertical tick marks on the *x* axis indicate the mirror’s central point of intersection; in (*c*), the intensity FWHM limits are shown. Below each shape, the longitudinal positions and widths of the 17 actuators are shown, staggered for clarity. The fitting r.m.s. differences are shown below each fit.

Three types of static fitting are applied to each of the three surface shapes, with the results shown in Fig. 5 ▸. The matrix of basis functions **A** is built from the calculated, individually actuated surface shapes described in Section 3. The fitting method of equation (1) assumes that the shapes scale linearly, they are independent (*i.e.* not coupled), and they are able to move accurately according to the applied voltage.

In Fig. 5 ▸(*a*), we fit using 17 actuators plus constant offset and linear *tilt* terms, applying a uniform weighting across the full domain. Again with uniform weighting, the fits in Fig. 5 ▸(*b*) remove the constant offset term and include the downstream beam stabilization described by equation (2). Fits in Fig. 5 ▸(*c*) use a weighting based on a Gaussian intensity profile with a FWHM of 120 mm, centered on the central point of intersection. To optimize the Strehl ratio of a coherent beam, we use the electric-field amplitude (square root of intensity) as the weighting. This weighting is effectively still Gaussian, but with a FWHM of 240 mm.

Results for Shapes 2 and 3 show that the fitting diverges from the target shape most on the left side, outside of the actuator region, as expected. Furthermore, the beam-stabilized solutions, Fig. 5 ▸(*b*), more poorly track the target shape on the left side owing to the one fewer degree of freedom. However, they do track the shape closely in the center and the right side. In Fig. 5 ▸(*c*), the weighted fit concentrates the region of highest fitting fidelity at the center, at the expense of the edges.

Since the solutions are linear, the r.m.s. fitting error (uniform or weighted) for a given shape will scale in proportion to the r.m.s. magnitude of that shape.

Piezo-bimorph mirrors may be subject to a physical constraint on the voltage difference between adjacent channels. This is a safety measure intended to avoid opposing forces in close proximity that may damage the mirror. (The limit set by the manufacturer could be a few hundred volts, in practice.) This effectively limits the amplitude of the third derivative of the shape because it affects the change in local curvature that can be applied over short distances. In the examples shown in Fig. 5 ▸, the maximum voltage values applied in the fitting fall within ± 4.5 V, with the largest adjacent-channel difference being close to 9.0 V, well within the safe operating range.

## 5. Resonance

Mechanical stability is an essential property for X-ray mirrors. Effective optical systems must be designed with first natural frequencies well above the excitation frequencies of vacuum pumps, water-cooling lines and similar influences. With its uniform, rectangular cross section, the thickness and length of the cantilevered mirror determine the first resonant frequency. Neglecting the thin piezoceramic strips, this dependence can be approximated by (Blevins, 2001 ▸), 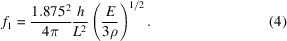
Here, *h* is the mirror thickness (14 mm), *L* is the free length (260 mm), ρ is the density of Si (2330 kg m^−3^) and *E* is Young’s modulus for bending out of plane (130 GPa). For our geometry, this resonant frequency is approximately 250 Hz.

## 6. Discussion

Modeling and mathematical analysis demonstrate that surface shape control can be achieved by adaptive X-ray mirrors mounted in a cantilevered geometry. Where applicable, this geometry simplifies the mount engineering, relieving concerns about distortions caused by differential thermal expansion and clamping mechanisms. While AXO mirrors with 1D arrays of piezoceramic actuators are now being included in X-ray beamlines, the cantilevered mounting geometry is unusual and, to date, untested in this application.

Both flat and curved mirrors can be engineered to be adaptive. In typical applications, where the required shape changes are less than or comparable with a micron of surface deformation, the beam footprint changes little during actuation. A linear approach to surface shape control may be applied where the change induced by the actuators is treated as perturbations to the inherent design. (More sophisticated analyses can be included where necessary.)

Focusing on the bending properties in isolation of other effects, our modeling shows how these mirrors can achieve arbitrary target shapes, similar to other published AXO mounting geometries (Colldelram *et al.*, 2017 ▸; Ichii *et al.*, 2019 ▸; Sutter *et al.*, 2022 ▸). The cantilever mount enables the mirror to bend freely, relative to the constraint on one side.

A unique aspect of the cantilever mounting is that actuation can lead to an asymmetric displacement and tilt of the mirror surface. This effect is most significant from the actuators closest to the mount, which cause the rest of the mirror to swing out of plane. With the inclusion of an external pitch mechanism in the mirror mount (to vary the incidence angle), the wavefront can be controlled and the lateral beam position can be stabilized downstream. We note that even for mirrors on fixed mounts (*e.g.* touching the Airy or Bessel points), surface shape changes will displace the mirror in the central and edge regions, leading to beam position shifting if it is not compensated.

Static modeling shows that for wavefront errors of low spatial frequency and 10 nm r.m.s. magnitude, surface fitting achieves fidelity within a fraction of a nm in the most important, central portions of the mirror, and on the unsupported end. Shape control is most challenging near the mounted end owing to the greater influence those actuators have on the position and angle of the rest of the surface. Dynamic shape control requires a more sophisticated approach, including optimization over time (Alcock *et al.*, 2019 ▸; Gunjala *et al.*, 2023 ▸), but static analysis can reveal the range of shapes that can be reached.

## Figures and Tables

**Table 1:** Materials properties used in the finite-element model

Property	Value	Reference
Silicon†		(Hopcroft *et al.*, 2014 ▸)
Young’s modulus, *E*_2_	130 GPa	
Poisson’s ratio, σ	0.28	

PZT‡		(Fujicera, 2022 ▸)
Young’s modulus, 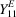	82 GPa	
Young’s modulus, 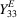	66 GPa	
Young’s modulus, 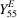	26 GPa	
Poisson’s ratio, σ	0.29	
Piezo charge constant, *d*_31_	−135 pm V^−1^	
Piezo charge constant, *d*_33_	310 pm V^−1^	
Piezo charge constant, *d*_15_	510 pm V^−1^	
Dielectric constant, 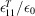	1590	
Dielectric constant, 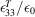	1470	

† In this 1D surface deflection analysis, the anisotropic properties of silicon are inconsequential.

‡ PZT—Pb(ZrTi)O_3_, lead zirconate titanate material C-213.

## Data Availability

The data and method (in a Python notebook) that support the findings of this study are available from the corresponding author upon reasonable request.
